# Diseño y validez de contenido del cuestionario continuidad del cuidado entre niveles asistenciales* **

**DOI:** 10.15649/cuidarte.2773

**Published:** 2023-09-08

**Authors:** Alexandra Marín Sánchez, María del Carmen Gogeascoechea-Trejo, Consuelo Vélez Álvarez, María Sobeida Leticia Blázquez-Morales, Patricia Pavón-León, María Gabriela Nachón-García, Dulce María Cinta Loaiza

**Affiliations:** 1 Programa de Doctorado en Ciencias de la Salud. Instituto de Ciencias de la Salud, Universidad Veracruzana, Xalapa - México. E-mail: alexandramarinpyp@gmail.com Universidad Veracruzana Instituto de Ciencias de la Salud Universidad Veracruzana Xalapa Mexico alexandramarinpyp@gmail.com; 2 Investigadora. Instituto de Ciencias de la Salud, Universidad Veracruzana, Xalapa -México. E-mail: cgogeascoechea@uv.mx Universidad Veracruzana Instituto de Ciencias de la Salud Universidad Veracruzana Xalapa Mexico cgogeascoechea@uv.mx; 3 Docente - investigadora. Departamento de Salud Pública, Universidad de Caldas, Manizales - Colombia. E-mail: consuelo.velez@ucaldas.edu.co Universidad de Caldas Departamento de Salud Pública Universidad de Caldas Manizales Colombia consuelo.velez@ucaldas.edu.co; 4 Investigadora. Instituto de Ciencias de la Salud, Universidad Veracruzana, Xalapa -México. E-mail: sblazquez@uv.mx Universidad Veracruzana Instituto de Ciencias de la Salud Universidad Veracruzana Xalapa Mexico sblazquez@uv.mx; 5 Investigadora. Instituto de Ciencias de la Salud, Universidad Veracruzana, Xalapa -México. E-mail: ppavon@uv.mx - Universidad Veracruzana Instituto de Ciencias de la Salud Universidad Veracruzana Xalapa Mexico ppavon@uv.mx; 6 Investigadora. Instituto de Ciencias de la Salud, Universidad Veracruzana, Xalapa -México. E-mail: gnachon@uv.mx Universidad Veracruzana Instituto de Ciencias de la Salud Universidad Veracruzana Xalapa Mexico gnachon@uv.mx; 7 Investigadora. Instituto de Salud Pública, Universidad Veracruzana, Xalapa - México. E-mail: dcinta@uv.mx Universidad Veracruzana Instituto de Salud Pública Universidad Veracruzana Xalapa Mexico dcinta@uv.mx

**Keywords:** Calidad de la Atención de Salud, Continuidad de la Atención al Paciente, Cuidado de Transición, Estudio de Validación, Revisión por Pares, Quality of Health Care, Continuity of Patient Care, Transitional Care, Validation Study, Peer Review, Qualidade da Assistencia a Saúde, Continuidade da Assistencia ao Paciente, Cuidado Transicional, Estudo de Validagao, Revisao por Pares

## Abstract

**Introducción:**

La falta de continuidad del cuidado puede ocasionar omisiones o duplicaciones en las acciones dirigidas al cuidado de usuarios con Enfermedades Crónicas No Transmisibles (ECNT), generando un posible deterioro de su salud. Particularmente, en México y Colombia no existe un instrumento que evalúe la continuidad del cuidado que incluya sus tres elementos esenciales.

**Objetivos:**

Diseñar un instrumento que evalúe la continuidad del cuidado entre niveles asistenciales en usuarios con ECNT en México y Colombia; y validar el contenido del cuestionario por medio de un juicio de expertos en versiones adaptadas al contexto mexicano y colombiano.

**Materiales y Métodos:**

Se diseñó el cuestionario Continuidad del Cuidado entre Niveles Asistenciales. Se realizó el proceso de validación de contenido por expertos usando el método Delphi. Se seleccionaron 16 jueces expertos (8 por país). Los ítems del cuestionario fueron evaluados bajo cuatro categorías: suficiencia, claridad, coherencia y relevancia. Se realizaron dos rondas de evaluación para determinar el grado de concordancia entre jueces.

**Resultados:**

El cuestionario obtuvo un Coeficiente de Validez de Contenido General “Excelente” para ambos países (0,97). La versión final quedó conformada por 85 ítems divididos en tres secciones.

**Discusión:**

Este instrumento, a diferencia de otros, evalúa desde la experiencia de los usuarios con ECNT la continuidad del cuidado de forma multidisciplinaria en los tres niveles de atención.

**Conclusión:**

El cuestionario alcanzó una validez de contenido esperada usando el método Delphi, para evaluar la continuidad del cuidado entre niveles asistenciales en usuarios con ECNT según el contexto mexicano y colombiano.

## Abstract (en)

**Introduction:**

In the absence of continuity of care, actions aimed at the care of users with chronic non communicable diseases (NCDs) may be omitted or duplicated, which can potentially worsen users' health. In Mexico and Colombia, in particular, there is no instrument for assessing continuity of care that includes its three essential elements.

**Objective:**

To develop an instrument to assess care continuity across levels of care for users with NCDs in Mexico and Colombia and validate the content of the questionnaire through expert judgment of versions adapted to the Mexican and Colombian contexts.

**Materials and Methods:**

The Continuity of Care across Levels of Care Questionnaire was designed. The content validation process was carried out by experts using the Delphi technique. Sixteen experts were selected (8 per country). Questionnaire items were assessed in four categories: sufficiency, clarity, coherence, and relevance. Two assessment rounds were conducted to determine the level of experts' agreement.

**Results:**

The questionnaire obtained an ‘Excellent' overall Content Validity Coefficient in both countries (0.97).

**Discussion:**

This instrument, unlike others, assesses the continuity of care in a multidisciplinary manner across the three levels of care from the experience of users with NCDs.

**Conclusion:**

The questionnaire achieved the expected content validity using the Delphi technique to assess care continuity across levels of care for users with NCDs, according to the Mexican and Colombian contexts.

## Resumo

**Introdugáo:**

A falta de continuidade do cuidado pode ocasionar omissóes ou duplicidades nas agóes voltadas para o atendimento aos usuários com Doengas Crónicas Nao Transmissíveis (DCNT), gerando um possível agravamento de sua saúde. Particularmente, no México e na Colómbia nao existe um instrumento que avalie a continuidade do cuidado que inclua seus tres elementos essenciais.

**Objetivo:**

Desenhar um instrumento que avalie a continuidade do cuidado entre os níveis de atengao em usuários com DCNT no México e na Colómbia; e validar o conteúdo do questionário por meio de julgamento de especialistas em versóes adaptadas ao contexto mexicano e colombiano.

**Materiais e Métodos:**

Foi elaborado o questionário de Continuidade de Cuidados entre os Níveis de Cuidados. O processo de validagao de conteúdo foi realizado por especialistas por meio do método Delphi. Foram selecionados 16 juízes especialistas (8 por país). Os itens do questionário foram avaliados em quatro categorias: suficiencia, clareza, coerencia e relevancia. Duas rodadas de avaliagao foram realizadas para determinar o grau de concordancia entre os juízes.

**Resultados:**

O questionário obteve um Coeficiente de Validade de Conteúdo Geral "Excelente" para ambos os países (0,97). A versao final foi composta por 85 itens divididos em tres segóes.

**Discussao:**

Este instrumento, diferente de outros, avalia a partir da experiencia dos usuários com DCNT a continuidade do cuidado de forma multidisciplinar nos tres níveis de atengao.

**Conclusao:**

O questionário atingiu a validade de conteúdo esperada usando o método Delphi, para avaliar a continuidade do cuidado entre níveis de atengao em usuários com DCNT de acordo com o contexto mexicano e colombiano.

## Introducción

La Organización Mundial de la Salud (OMS) y asociaciones científicas en salud han reconocido a la continuidad del cuidado como parte primordial en la calidad asistencial al enfocar la atención centrada en la persona, mejorar su calidad de vida y promover su bienestar^1^. La Comisión Conjunta de Acreditación de Organizaciones Sanitarias; organización internacional encargada de acreditar y certificar servicios médicos en el mundo con rigurosos parámetros de seguridad y calidad en la atención del paciente; define a la continuidad del cuidado como "... el grado en que la asistencia que necesita el paciente está coordinada eficazmente entre diferentes profesionales y organizaciones con relación al tiempo”^2^.

En 2016 un estudio realizado en México propone que la continuidad del cuidado la conforman tres elementos esenciales^3^: a) personas: referente al usuario, su cuidador y el profesional de la salud; b) entorno: lugar donde se generan las necesidades de cuidado y se organiza la atención; y c) intervenciones de la continuidad del cuidado o cuidadoras: que pueden ser de tipo informacional, relacional y de gestión^3^^-^^5^.

Las personas diagnosticadas con Enfermedades Crónicas No Transmisibles (ECNT) requieren de un cuidado centrado en la persona, integral, coordinado y continuo entre niveles de atención por parte de un equipo multidisciplinario^6^^,^^7^^,^^8^, con el fin de mantener el control de su enfermedad y prevenir complicaciones. Por consiguiente, la continuidad del cuidado coadyuva a la articulación de los servicios^9^, disminuye los reingresos hospitalarios, aumenta la satisfacción en la atención, así como fortalece la comunicación entre usuarios y el personal de salud^10^^,^^11^. No obstante, la falta de continuidad del cuidado puede ocasionar omisiones, duplicaciones o contradicciones^1^ en las decisiones y acciones dirigidas al cuidado de los usuarios con ECNT, generando un posible deterioro de su estado de salud^12^.

Existen algunos instrumentos que miden la calidad de la continuidad del cuidado a través de las actividades realizadas por el personal de enfermería^12^ como planes de egreso hospitalario^13^ o informes de continuidad del cuidado^14^, algunos determinan el nivel de satisfacción de los usuarios después de su hospitalización^15^; sin embargo, la estandarización de estos instrumentos para su uso no es fácil, por la variabilidad de los sistemas de salud latinoamericanos y no se evidenció un instrumento que evaluara de manera integral la continuidad del cuidado.

México y Colombia no están exentos de contar con un instrumento adaptado a su contexto y que evalúe la continuidad del cuidado con sus tres elementos esenciales en la atención de usuarios con ECNT. A pesar de que ambos países poseen sistemas de salud diferentes, fueron seleccionados en este estudio por implementar estrategias y políticas públicas dirigidas a prevenir y controlar las ECNT; además de que cuentan con una estructura organizacional similar para la atención en salud de estos usuarios.

En consecuencia, es trascendental diseñar y validar un instrumento que explore - desde la experiencia de los usuarios con ECNT- la continuidad del cuidado brindada por un equipo multidisciplinario con énfasis en reconocer las actividades derivadas de esta atención dirigidas al cuidado de la salud, en los distintos niveles asistenciales de los servicios públicos de salud de México y Colombia, con el fin de aportar a la comunidad científica y a los tomadores de decisiones de cada país, un instrumento válido que permita contribuir a la comprensión de los procesos de atención en salud en usuarios con enfermedades crónicas y generar evidencia científica para establecer procesos de mejora continua de la calidad de la atención en salud de estos usuarios.

Por lo anterior, los objetivos de este estudio fueron:

1) diseñar un instrumento que evalúe la continuidad del cuidado entre niveles asistenciales en usuarios diagnosticados con ECNT que asisten a servicios públicos de salud en México y Colombia 2) validar el contenido del cuestionario por medio de un juicio de expertos en versiones adaptadas al contexto mexicano y colombiano.

## Materiales y Métodos

Tipo de estudio: Diseño metodológico para la validación de contenido de instrumentos utilizando el método Delphi.

Diseño del cuestionario: Se diseñó el instrumento denominado “Continuidad del Cuidado Entre Niveles Asistenciales (CCUNA)” para conocer la experiencia de continuidad del cuidado entre niveles asistenciales a través de sus tres elementos esenciales: personas, entorno e intervenciones cuidadoras, en usuarios con ECNT sin seguridad social en Xalapa (México) y sin capacidad de pago en Manizales (Colombia).

Su construcción se inició luego de una revisión bibliográfica sobre la temática, sumado al aporte de la experiencia profesional de los autores; de igual manera, se fundamentó principalmente en el diseño y estructura del instrumento CCAENA®^16^, elaborado para evaluar la continuidad asistencial entre niveles de atención desde la perspectiva de los usuarios, el cual fue adaptado lingüísticamente para seis países latinoamericanos, entre ellos México y Colombia^17^^,^^18^. Aun cuando CCAENA® es un instrumento de uso libre, que está a disposición pública en la página web del proyecto Equity-LA II (https://www2.equity-la.eu/es/index.php), se solicitó autorización a los autores para tomarlo como base para diseñar el cuestionario validado en este estudio.

Después de este proceso, el cuestionario CCUNA quedó conformado por tres secciones: 1) personas: datos sociodemográficos y, datos de la enfermedad crónica y autoconcepto de salud, 2) experiencias de la continuidad del cuidado entre niveles asistenciales: entorno a nivel asistencial y entorno familiar e, 3) intervenciones de la continuidad del cuidado o cuidadoras: intervención de tipo informacional, relacional y gestión. Inicialmente la primera versión incluyó 133 ítems, que en su mayoría tenían respuesta abiertas, en escala dicotómica (sí, no) y tipo Likert (siempre, muchas veces, pocas veces, nunca).

Para evaluar la validez de contenido del cuestionario CCUNA, se utilizó el método Delphi^19^ siguiendo tres fases utilizadas por García y Suarez^20^ que fueron adaptadas para esta investigación:

### 1. Fase de preparación

*Selección de jueces expertos:* Según lo indicado en la literatura^21^^,^^22^ el número de jueces expertos adecuado para conformar un grupo de evaluación de un instrumento debe ser mayor a dos; por consiguiente, para este estudio se decidió integrar tanto para México como para Colombia un grupo de ocho jueces, los cuales debían cumplir con los siguientes criterios de selección: 1) haber cursado estudios de posgrado; 2) tener experiencia profesional de mínimo cuatro años en el área de ciencias de la salud; 3) formar parte en una línea de investigación que aborde temas del cuidado de la salud, continuidad del cuidado o ECNT; 4) trabajar en una institución de prestación de servicios de salud, educación superior o instituto de investigación en México o Colombia y; 5) poseer un índice h igual o superior a 5 (medición de la calidad profesional de científicos, en función de la cantidad de citas que han recibido sus artículos). A cada uno de los posibles candidatos, se le envió vía correo electrónico una invitación formal para formar parte como juez, se adjuntó la carta de consentimiento informado y, se explicó el objetivo e instrucciones del proceso de validación de contenido del cuestionario CCUNA, detallando que su participación sería anónima y protegiendo su identidad en caso de aceptar ser parte del grupo de jueces expertos.

*Vía de consulta:* el correo electrónico fue el medio de comunicación utilizado con cada integrante del grupo de jueces expertos por país durante el proceso de validación.

### 2. Fase de consulta

*Validación de contenido:* Aquellos investigadores que aceptaron participar como jueces en el proceso de validación, les fue enviado por correo electrónico la primera versión del cuestionario CCUNA y un formato de validación de contenido con las instrucciones para evaluarlo. Dicho formato contenía los criterios de evaluación para la validez de contenido del instrumento propuestos por Escobar y Cuervo^22^, en el cual los ítems que conformaban cada sección del cuestionario debían ser evaluados bajo cuatro categorías: suficiencia, claridad, coherencia y relevancia. Cada categoría fue calificada por medio de una escala tipo Likert de 1 a 4 puntos, donde el valor de 1 corresponde a: no cumple con el criterio; 2: bajo nivel de cumplimiento; 3: moderado nivel de cumplimiento y; 4: alto nivel de cumplimiento. Además, se incorporó un espacio al final de cada ítem donde los expertos podían indicar sus observaciones y recomendaciones en caso de que fuera necesario.

Para lograr el tiempo estimado de validación del instrumento, a los jueces se les notificó tener 15 días naturales para su evaluación.

*Rondas de consulta:* Se planteó realizar las rondas necesarias hasta establecer a un consenso entre los jueces expertos por país.

*Procesamiento estadístico:* Después de obtener los resultados de las calificaciones realizadas por los jueces en cada ronda, en el formato de validación de contenido del cuestionario CCUNA, se calculó el Coeficiente de Validez de Contenido (CVC) propuesto por Hernández-Nieto^23^, que evalúa el grado de acuerdo de los evaluadores por cada ítem y del cuestionado en forma global^23^^,^^24^. Para calcular el CVC de cada ítem, fue necesario aplicar las siguientes fórmulas:




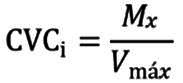




Donde M_v_ fue la media de las calificaciones de cada ítem dadas por los jueces y V_max_, fue el valor máximo posible que pudiese obtener cada ítem. De igual forma, se debió calcular el Pe_i_ que fue la probabilidad de error de cada ítem, reduciendo el posible sesgo de los jueces, al emplear la siguiente fórmula:




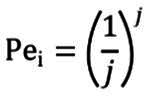




Donde j fue el número de jueces participantes. Posteriormente, con los anteriores resultados se pudo calcular el CVC aplicando la siguiente fórmula:




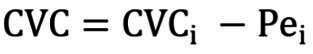




Para la interpretación de los resultados, se clasificó cada CVC en tres valores posibles: mayor de 0,90 “Excelente”; mayor de 0,80 hasta 0,90 “Bueno” y; menor o igual a 0,80, “Deficiente”.

Los ítems calificados con un CVC “Deficiente” se eliminaron o modificaron de acuerdo con las recomendaciones realizadas por de los jueces expertos.

Finalmente, para calcular el CVC Global (CVCG) se obtuvo la media de la sumatoria de los CVC de todos ítems, esperando alcanzar un valor mayor a 0,80 en cada evaluación del cuestionario CCUNA para obtener finalmente una versión definitiva para México y Colombia. Los datos se analizaron en el paquete estadístico IBM SPSS versión 26.0. Las bases de datos para calcular el CVC y el CVCG del cuestionario CCUNA en cada ronda para México y Colombia fueron almacenadas en Mendeley Data^25^.

### 3. Fase de consenso

*Construcción del consenso:* Cuando se alcanzó un valor mayor a 0,80 en el CVC y CVCG se determinó el grado de acuerdo en cada grupo de jueces expertos por país.

*Reporte de resultados:* Se elaboró un informe final del proceso de validación de contenido y junto con éste se envió por correo electrónico un certificado de participación a cada juez experto.

Elaboración final del cuestionario: Una vez realizados los ajustes correspondientes a las sugerencias y recomendaciones emitidas por los dos grupos de jueces expertos, quedó elaborada la versión final del cuestionario CCUNA sin alterar la validez de contenido.

## Resultados

El proceso de validación de contenido se desarrolló en dos rondas entre octubre de 2020 y marzo de 2021.

### Selección de jueces expertos participantes por país

En la primera ronda de validación, en México de los 16 investigadores contactados que cumplieron con los criterios de selección, ocho (50%) aceptaron formar parte del grupo de jueces; en Colombia de los 28 investigadores contactados que cumplieron con los criterios de selección, ocho (28,6%) aceptaron participar.

En la segunda ronda, de los 16 jueces expertos de ambos países contactados en la primera ronda, se continuó con la participación de cinco jueces mexicanos (62,5%) y cuatro jueces colombianos (50%); en consecuencia, se debió buscar bajo los mismos criterios de selección a tres nuevos jueces mexicanos y cuatro jueces colombianos para completar los ocho jueces expertos por país. Las razones por las cuales los tres jueces mexicanos (37,5%) y los cuatro jueces colombianos (50%) desistieron continuar como participantes fueron: tiempo insuficiente por carga académica (100% México; 75% Colombia) e incapacidad laboral por enfermedad (25% Colombia).

### Caracterización de los jueces participantes por país

En la primera ronda, el grupo de jueces mexicanos se conformó en igual proporción de hombres (50%) y mujeres (50%); mientras que el grupo de jueces colombianos se conformó en su totalidad por mujeres (100%); la edad promedio fue de 44±12,3 años para México y 51±9,9 años para Colombia. En ambos grupos de jueces el 62,5% tenían como último grado de estudios un doctorado; más de la mitad tenía experiencia en el área de enfermedades crónicas (50% México; 62,5% Colombia), con una experiencia profesional de 19±9 y 25±9,1 años de respectivamente. Además, el 62,5% de los jueces mexicanos y el 75% de los jueces colombianos trabajaban en instituciones educativas como docente e investigador y el 50% de los jueces mexicanos tenían un índice h de 5, en tanto que, el 37,5% de los jueces colombianos fue de 6 (Tabla 1).

En la segunda ronda, el 75% de los jueces mexicanos y el 100% de jueces colombianos fueron mujeres; la edad promedio fue de 47±12,1 y 53±8,8 años respectivamente. El 50% de los jueces mexicanos tenían un doctorado como último grado de estudios, mientras que, el 62,5% de los jueces colombianos tenían estudios de maestría; el 62,5% de los jueces de ambos países tenían experiencia en el área del cuidado de la salud, con 22±10,9 y 24±7 años de experiencia profesional respectivamente, el 62,5% en ambos grupos laboraban en una institución educativa como docente e investigador y el 37,5% de los jueces mexicanos tenían un índice h de 6, mientras que, el 50% de los jueces colombianos fue de 5 (Tabla 1).

**Tabla 1:** Caracterización de los jueces participantes por país

Característica	Primera ronda		Segunda ronda	
	México n=8	Colombia n=8	México n=8	Colombia n=8
Sexoa				
Mujer	4 (50,0)	8 (100,0)	6 (75,0)	8 (100,0)
Hombre	4 (50,0)	0 (0,0)	2 (25,0)	0 (0,0)
Edad b	44 ± 12,3	51 ± 9,9	47 ± 12,1	53 ± 8,8
Último grado de estudios^a^				
Maestría	3 (37,5)	3 (37,5)	3 (37,5)	5 (62,5)
Doctorado	5 (62,5)	5 (62,5)	4 (50,0)	3 (37,5)
Post - doctorado	0 (0,0)	0 (0,0)	1 (12,5)	0 (0,0)
Áreas de experiencia académica o profesionala				
Enfermedades crónicas	4 (50,0)	5 (62,5)	4 (50,0)	5 (62,5)
Cuidado de la salud	4 (50,0)	4 (50,0)	5 (62,5)	5 (62,5)
Investigación	4 (50,0)	2 (25,0)	5 (62,5)	2 (25,0)
Otros^c^	5 (62,5)	3 (37,5)	6 (75,0)	3 (37,5)
Años de experiencia profesional^b^	19 ± 9,0	25 ± 9,1	22 ± 10,9	24 ± 7,0
Cargo^a^				
Docente e investigador (a)	5 (62,5)	6 (75,0)	5 (62,5)	5 (62,5)
Otros	3 (37,5)	2 (25,0)	3 (37,5)	3 (37,5)
Característica	Primera ronda		Segunda ronda	
	México n=8	Colombia n=8	México n=8	Colombia n=8
Índice h^a^				
5	4 (50,0)	1 (12,5)	2 (25,0)	4 (50,0)
6	0 (0,0)	3 (37,5)	3 (37,5)	3 (37,5)
7	0 (0,0)	2 (25,0)	0 (0,0)	1 (12,5)
8	1 (12,5)	0 (0,0)	1 (12,5)	0 (0,0)
9	1 (12,5)	1 (12,5)	1 (12,5)	0 (0,0)
10	0 (0,0)	0 (0,0)	2 (25,0)	0 (0,0)
12	1 (12,5)	0 (0,0)	1 (12,5)	0 (0,0)
13	1 (12,5)	0 (0,0)	0 (0,0)	0 (0,0)
22	0 (0,0)	1 (12,5)	0 (0,0)	0 (0,0)

*a Los datos se representan como n (%) b Los datos se representan como media (± desviación estándar) c Otros = incluye respuesta < 3%*

### Resultados del Coeficiente de Validez de Contenido

En la primera ronda se evaluaron 133 ítems: en el grupo de jueces expertos mexicanos, el 49,62% de los ítems lograron un CVC “Bueno” y un 30,82% “Deficiente”; mientras que, en el grupo de jueces expertos colombianos, el 75,18% de los ítems evaluados logró un CVC “Excelente” y el 21,80% “Bueno”. El CVCG fue calificado como “Bueno” (0,83) por el grupo de jueces expertos mexicanos y “Excelente” (0,94) por el grupo de jueces expertos colombianos (Tabla 2).

En la segunda ronda se evaluaron 86 ítems: en ambos grupos, el 98,83% de los ítems alcanzó un CVC “Excelente” y el 1,16% de los ítems logró un CVC “Bueno”. Ningún ítem fue evaluado como “Deficiente”. El CVCG fue calificado como “Excelente” (0,97) en ambos grupos de jueces expertos mexicanos y colombianos (Tabla 2).

**Tabla 2:** Resultados del Coeficiente de Validez de Contenido por ronda

Resultados del CVC^a^	Primera ronda		Segunda ronda	
	México	Colombia	México	Colombia
Ítems contenidos en el instrumento	n=133	n=133	n=86	n=86
Ítems con CVC Excelente (>0,90)	26 (19,54)	100 (75,18)	85 (98,83)	85 (98,83)
Ítems con CVC Bueno (>0,80 y <=0,90)	66 (49,62)	29 (21,80)	1 (1,16)	1 (1,16)
Ítems con CVC Deficiente (<=0,80)	41 (30,82)	4 (3,00)	0 (0,00)	0 (0,00)
CVC General	0,83	0,94	0,97	0,97

*a Los datos se representan como n (%)*

### Ítems evaluados por ronda de validación

De los ítems evaluados en la primera ronda (133) el 33,83% se conservó; el 30,82% se modificó su redacción según las recomendaciones u observaciones emitidas por los jueces expertos y; el 35,33% se eliminó por considerarse no pertinente, duplicado o inconsistente (Tabla 3).

De los ítems evaluados en la segunda ronda (86), el 75,58% fue conservado, el 23,25% fue modificado en su redacción según las observaciones y recomendaciones indicadas en ambos grupos de jueces. Por lo que no hubo diferencias en el criterio de evaluación y el 1,16% fue eliminado al ser fusionado con otro ítem. Finalmente, el cuestionario quedo conformado por 85 ítems (Tabla 3).

**Tabla 3:** Ítems evaluados por ronda de validación

Rondas de validación	Ítems evaluados	Ítems conservados^a^	Ítems modificados^a^	Ítems eliminados^a^
Primera	133	45 (33,83)	41 (30,82)	47 (35,33)
Segunda	86	65 (75,58)	20 (23,25)	1 (1,16)

*a Los datos se representan como n (%)*

### Conformación final del instrumento

Al concluir el proceso validación de contenido, el cuestionario CCUNA quedó conformado por 85 ítems divididos en tres secciones: 1) Personas (15 ítems), 2) Experiencias de la continuidad del cuidado entre niveles asistenciales (31 ítems) e 3) Intervenciones de la continuidad del cuidado o cuidadoras (39 ítems). Las versiones del cuestionario adaptadas al contexto mexicano y colombiano quedaron conformadas por el mismo número de ítems e incluyen términos propios de cada país (Tabla 4 y Anexo 1).

La versión final del cuestionario puede ser solicitada al autor corresponsal (o puede consultar material complementario).

**Tabla 4:** Conformación final del instrumento

Sección	Dimensión	Número de ítems
Sección 1:	1. Datos sociodemográficos	12
Personas	2. Datos de la enfermedad crónica y autoconcepto de salud	3
Sección 2:	3. Entorno	
Experiencias de la continuidad	-Entorno de la continuidad del cuidado: nivel asistencial	26
del cuidado entre niveles asistenciales	-Entorno de la continuidad del cuidado: familiar	5
Sección 3:	4. Intervención de tipo informacional	16
Intervenciones de la continuidad	5. Intervención de tipo relacional	14
del cuidado o cuidadoras	6. ntervención de gestión	9
Total de ítems:		85

## Discusión

La utilización del método Delphi para la validación de contenido del cuestionario CCUNA permitió alcanzar el consenso entre las diversas opiniones emitidas por los jueces participantes expertos en el cuidado de la salud y ECNT de México y Colombia, confirmando lo que se resalta en otras publicaciones como un método sistemático y flexible para evaluar instrumentos de diferentes áreas del conocimiento y contextos^26^^,^^27^; no obstante, existen otros procesos utilizados para la validación de contenido^22^ que deben ser examinados detalladamente al momento de ser seleccionados.

Para el diseño del cuestionario CCUNA, previo al inicio de proceso de validación de contenido, se realizó una revisión de la literatura en la que se encontraron diferentes instrumentos que evalúan la calidad de la continuidad del cuidado como lo son el CTM-15 y CTM-3 (Care Transitions Measure) que utilizan el Informe de Continuidad de Cuidados para determinar el nivel de satisfacción de los usuarios después de su hospitalización^28^, el PACIC (Patient Assment of Chronic Illness) que evalúa la atención de enfermedades crónicas y el apoyo para su automanejo desde la perspectiva del paciente^29^, el cuestionario de continuidad de Nijmegen (NCQ-N) que mide la continuidad de la atención experimentada por los pacientes en múltiples entornos de atención e independientemente de la morbilidad^30^ y el cuestionario de Continuidad Asistencial Entre Niveles (CCAENA®)^16^^-^^18^ diseñado para evaluar este proceso desde la perspectiva de los usuarios apoyado en la atención médica, mismo que fue seleccionado en este estudio por aproximarse a los conceptos de la continuidad asistencial y continuidad del cuidado.

El cuestionario construido en este estudio, a pesar de estar basado principalmente en el diseño y estructura del instrumento CCAENA®, tiene la particularidad de evaluar desde la experiencia de los usuarios con ECNT la continuidad de sus cuidados en el primer, segundo y tercer nivel de atención, enfocado a una atención multidisciplinaria, haciendo énfasis en las actividades derivadas de esta atención y en la información, recomendaciones e indicaciones sobre el cuidado de la salud brindadas por los profesionales de la salud de cada nivel.

Lo anterior fue el resultado del apoyo de los jueces que participaron en este estudio al tener experiencia académica y profesional en las líneas de investigación enfocadas en el cuidado de la salud y enfermedades crónicas; así como haber cursado maestría o doctorado lo que brindó certeza de los resultados obtenidos^22^.

Estas características fueron similares con diversas publicaciones que resaltan la importancia de definir el perfil adecuado de los expertos, con el objetivo de conseguir evaluar de manera imparcial y obtener una retroalimentación que enriquezca la validación del instrumento^21^^,^^24^^,^^27^ y aunque no se encontró en la literatura una cantidad exacta de jueces expertos que debían participar en este proceso, se considera fundamental la selección de un número apropiado para proporcionar una confiabilidad en la validez de contenido de un instrumento^21^^,^^22^^,^^26^; en esta investigación se decidió conformar dos grupos de jueces con ocho expertos por país, coincidente con el rango de dos a treinta jueces que sugieren otros estudios^21^^,^^22^.

Para estimar la validez de contenido del cuestionario por juicio de expertos, fue necesario conocer el grado concordancia entre ellos^22^. Para ello se utilizó el Coeficiente de Validez de Contenido^23^^,^^24^, el cual calcula el grado de acuerdo por ítem entre varios expertos participantes en cada ronda según su experiencia en el abordaje de la temática tratada en éste. Algunos autores también proponen otras pruebas estadísticas como el Coeficiente de Kappa para obtener el grado de concordancia entre varios individuos, generalmente es usado en las ciencias biológicas y sociales; de igual manera, el Coeficiente de concordancia de W de Kendall, para conocer el grado de asociación o acuerdo entre diferentes evaluadores^22^^,^^31^.

En esta investigación después de analizar los resultados obtenidos, el consenso de los jueces se alcanzó en la segunda ronda de validación; otros autores han reportado realizar hasta tres rondas para lograr este consenso^19^^,^^32^. No obstante, es recomendable realizar entre dos a tres rondas como máximo para evitar que la validación sea una tarea larga, costosa y conlleve a un abandono por parte de los jueces expertos^33^.

Respecto con los resultados obtenidos en el CVC de cada uno de los ítems que conforman las tres secciones del cuestionario, no pudieron ser contrastados con otros instrumentos similares nacionales o internacionales, ni con los resultados obtenidos en el análisis de los ítems del CCAENA® ya que sus autores utilizaron diferentes propiedades psicométricas durante el proceso de validación (validez de constructo, consistencia interna, multidimensionalidad de las escalas y análisis de grupos conocidos)^16^^-^^18^ a las utilizadas en esta investigación (método Delphi y Coeficiente de Validez de Contenido).

Sin embargo, es importante señalar que para lograr un CVC mayor a 0,80 en todos los ítems del cuestionario CCUNA en sus dos versiones, fue necesario identificar aquellos que presentaron mayores discrepancias en los criterios de evaluación establecidos para la validez de contenido, siendo principalmente algunos de los ítems contenidos en la “Sección 2: Experiencias de la continuidad del cuidado entre niveles asistenciales” los que fueron evaluados con bajas calificaciones en las categorías de claridad y coherencia; lo anterior, implicó que fueran modificados en su redacción o eliminados según las recomendaciones indicadas por los jueces expertos antes de concluir el proceso de validación de contenido.

Por último, si bien este instrumento fue validado por los jueces expertos en México y Colombia para ser aplicado en servicios públicos de salud de estos países, también podría ser utilizado para realizar futuros estudios en otros servicios públicos o privados de salud de países latinoamericanos con contextos similares; siempre y cuando este sea sometido a un proceso de adaptación transcultural (para países donde el español es el idioma oficial) o por traducción y adaptación al contexto del país (para países con otro idioma), especialmente para evaluar la continuidad el cuidado entre niveles asistenciales en usuarios con otro tipo de diagnóstico médico o usuarios pertenecientes a otros programas de promoción de la salud y prevención de la enfermedad; por ejemplo, el programa de gestantes y recién nacido, donde se debe garantizar una atención integral y segura en todos los niveles^34^.

### Limitaciones

Durante el proceso de validación de contenido existió la deserción de algunos jueces, argumentando motivos personales o académicos para no continuar participando. Esto ocasionó buscar e incorporar nuevos jueces expertos y capacitarlos para reanudar la validación y evitar retrasos en la evaluación del instrumento.

## Conclusiones

El diseñar el cuestionario CCUNA permitió conocer la existencia de una estrecha relación en el concepto de continuidad asistencial y continuidad del cuidado, y evidenciar la poca claridad en el consenso de los elementos esenciales que la conforman; hallazgo que se evidenció en la literatura revisada y en el número de instrumentos encontrados sobre la temática. De igual manera, el validar el contenido del cuestionario CCUNA por medio del acuerdo entre los jueces expertos al obtener un CVC excelente usando el método Delphi, proporcionó un instrumento válido y fiable que permitirá evaluar la continuidad del cuidado (incluyendo sus tres elementos esenciales) entre niveles de atención en los usuarios diagnosticados con ECNT que asisten a los servicios públicos de salud de México y Colombia. Sin embargo, se recomienda continuar con los análisis que proporcionen más información de otras propiedades psicométricas (validez y confiabilidad interna) del instrumento.

## Anexo 1. Versión final del Cuestionario de Continuidad del Cuidado Entre Niveles Asistenciales -CCUNA-

**EI diseño de este documento fue basado en el Cuestionario de Continuidad Asistencial Entre Niveles de Atención (CCAENA©) del proyecto Equity-LA II (https://www2.equity-la.eu/es/index.php)*
